# New Ancient Egyptian Human Mummies from the Valley of the Kings, Luxor: Anthropological, Radiological, and Egyptological Investigations

**DOI:** 10.1155/2015/530362

**Published:** 2015-08-06

**Authors:** Frank Rühli, Salima Ikram, Susanne Bickel

**Affiliations:** ^1^Institute of Evolutionary Medicine, University of Zurich, 8057 Zurich, Switzerland; ^2^Department of Egyptology, American University of Cairo, Cairo 11835, Egypt; ^3^Egyptology, University of Basel, 4051 Basel, Switzerland

## Abstract

The Valley of the Kings (arab. *Wadi al Muluk*; KV) situated on the West Bank near Luxor (Egypt) was the site for royal and elite burials during the New Kingdom (ca. 1500–1100 BC), with many tombs being reused in subsequent periods. In 2009, the scientific project “The University of Basel Kings' Valley Project” was launched. The main purpose of this transdisciplinary project is the clearance and documentation of nonroyal tombs in the surrounding of the tomb of Pharaoh Thutmosis III (ca. 1479–1424 BC; KV 34). This paper reports on newly discovered ancient Egyptian human mummified remains originating from the field seasons 2010–2012. Besides macroscopic assessments, the remains were conventionally X-rayed by a portable X-ray unit *in situ* inside KV 31. These image data serve as basis for individual sex and age determination and for the study of probable pathologies and embalming techniques. A total of five human individuals have been examined so far and set into an Egyptological context. This project highlights the importance of ongoing excavation and science efforts even in well-studied areas of Egypt such as the Kings' Valley.

## 1. Introduction

The Valley of the Kings (arab.* Wadi al Muluk*; KV) situated on the West Bank near Luxor (Egypt) was the site for royal and elite burials during the New Kingdom (ca. 1500–1100 BC), with many tombs being subsequently reused by lesser elites (ca. 950–850 BC). Its remote and dry location helped for the preservation of the buried ancient human mummified remains [1–5]. The valley has been visited by robbers and tourists since antiquity; since the early 19th century AD, antiquarians and archaeologists have cleared and recorded tombs, with a total of 61 sepulchers being known by the start of the 20th century [6]. In 1912, The financier and excavator, Theodore Davis (1837–1915) famously declared the valley now “being exhausted” [7]. However, in late 1922, the archaeologist Howard Carter (1874–1939) and his colleagues discovered the now iconic tomb (KV 62) of Pharaoh Tutankhamun [8]. Since these days, almost one hundred years ago, discovering new tombs has become rare in the valley: in 2005, the Amenmesse Project found KV 63, an embalming cache [9], and in 2012, the University of Basel Kings' Valley Project found KV 64 [10–12]. Nowadays, most archeological research focuses on the documentation and precise recording of the hitherto known tombs and pits, reestablishing their precise location and analyzing their remaining contents.

**Figure 1:**
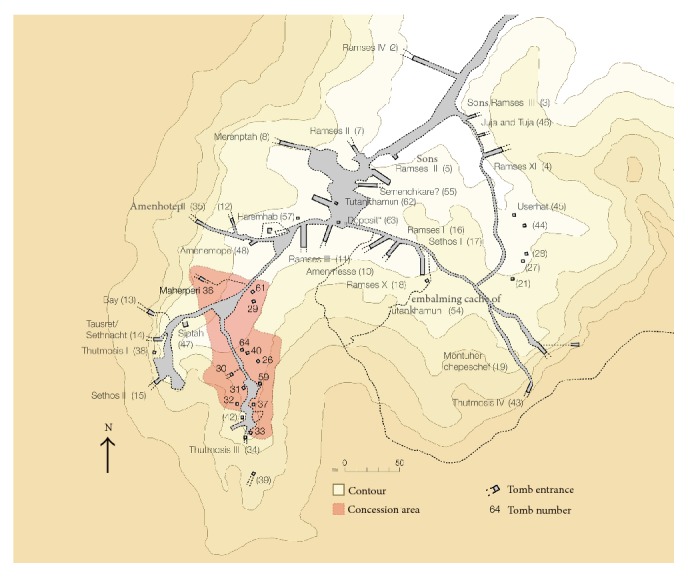
Map of the Kings' Valley with the concession area of the University of Basel Kings' Valley Project in red.

Researchers of the University of Basel (Switzerland) have been involved in Egyptological projects in the Kings' Valley since many years [13]. In 2009, the most recent scientific project “The University of Basel Kings Valley Project” (http://www.ubkvp.ch/; access date: 20 Dec. 2014) or (http://aegyptologie.unibas.ch/forschung/projekte/university-of-basel-kings-valley-project/; access date: 20 Dec. 2014) was started with Susanne Bickel as director and Elina Paulin-Grothe as field director. The main purpose of this transdisciplinary project is the investigation and documentation of nonroyal tombs in the surroundings of the tomb of Pharaoh Thutmose III (ca. 1450 BC; KV 34; Figure 1).

During the season of spring 2010, the project team started research on tomb KV 31, of which only the upper rim of the shaft was visible on the desert surface. No information about this tomb nor documentation of former archaeological explorations were known, although KV 31 has possibly been visited already by Giovanni Battista Belzoni (1778–1823) in 1817 and perhaps also by Victor Loret (1859–1946) or his team in 1898. As its shaft was entirely filled with sand and stones, it had not been surveyed by the Theban Mapping Project (http://www.thebanmappingproject.com/, for a first sketch of the tomb http://aegyptologie.unibas.ch/?id=21700, access date: Dec. 20, 2014).

**Figure 2:**
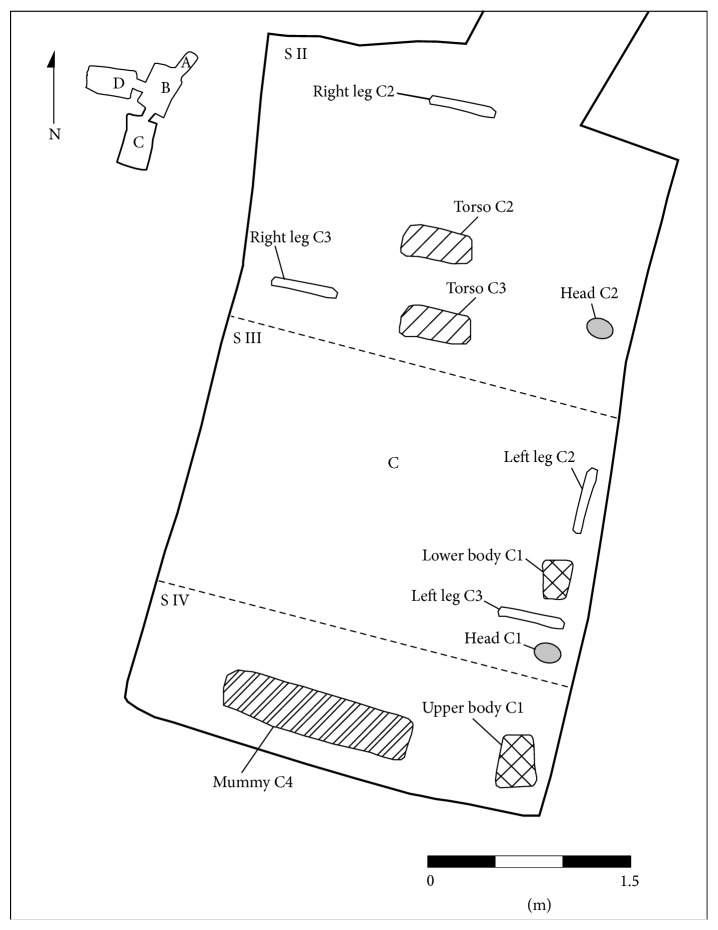
Room C of tomb KV 31 with the scattered fragments of mummy parts (survey: T. Alsheimer, University of Basel).

Tomb KV 31 lies on a steep slope on the west flank of the lateral valley; it consists of a vertical shaft with a depth of about 5 m, which gives access to a central room B (ca. 470 cm × 370 cm). The main burial chamber (room C, ca. 530 cm × 320 cm, Figure 2) lies to the south of the central room, a less properly cut room D lies to the west. The central room was filled in with a thick layer of desert debris possibly indicating a later reuse of the tomb. Rooms C and D contained the very fragmented remains of several burials, probably five individuals, which can be assigned to the mid-18th dynasty (ca. 1450–1400 BC, from the reign of Thutmose III to that of Amenhotep II) on the basis of a large quantity of pottery and some fragments of canopic jars. The original burials were severely looted in antiquity (21st dynasty, 11th–10th c. BC), some decades before the probable reuse of the tomb, and further damage by modern robbers seems assured. Robbers of all periods sought for valuables, mainly jewellery; ancient looters moreover retrieved all wooden objects for reuse. No wooden coffins remained in KV 31. The mummies of the individuals were stripped of all their bandages and violently disarticulated. Most of the mummies' remains were found clustered in room C (thus labelled 31.C), with one mummy found in room D (31.D). The remaining objects do not reveal the identity of the individuals. However, the quality of the fragmentary burial goods indicates their very high social status. During the mid-18th dynasty, the Kings' Valley was used as burial ground for members of the royal family and the kings' immediate entourage (queens, princesses, princes, wet-nurses, and royal companions, [14]). Future ancient DNA analyses might answer the question whether the individuals of KV 31 were related to each other and whether they belonged to the royal family or not.

The aim of this paper is to report on these ancient human mummified remains. The application of simple, on-site techniques (visual inspection, conventional X-rays) allows the investigators to reassemble the highly fragmented bodies as well as assess sex, individual age, and possible pre- and postmortem changes.

## 2. Material and Methods

All human remains found in KV 31 (chambers C and D), Valley of the Kings, Luxor, Egypt, were reassembled and analysed. The initial stage of analysis consisted of matching up body fragments to form complete individuals. The mummies and fragments thereof then were subjected to macroscopic examination by naked eye and magnifying glasses for mummification technology, taphonomic changes in the mummies, basic ageing and sexing, and identifying any particular lesions. This was finally followed by radiography. A total of 27 radiological images of all bodies were taken. Some of the images are of minor quality (field of view, exposure time); yet unfortunately no repetition of such lower quality images could be made due to local technical restrictions (traditional development over night only) and administrative restrictions prohibited the use of more modern and adequate radiographic equipment. The portable machine used was a Karmex Diagnostic X-ray Unit PX-20N (AC 115 V 50/60 HZ, 50–130 KVp 2–20 mA), together with Agfa industrial film. The kV was 60, 15 mAs, although there was some variation due to technical issues of electricity supply. The distance varied between 1.35 m and 1.45 m. The bodies were also photographed within the tomb. Ageing and sexing was based on standard anthropological criteria [15, 16] as far as possible based on the skeletonized, partially mummified, fully mummified, and in some cases even fully wrapped remains. After examination, the body parts were labelled and stored appropriately in individual modern coffins in KV 31.

## 3. Results

### 3.1. General Assessment and Macroscopic Appearance

First, an initial macroscopic assessment was undertaken. The various fragmented body parts, initially thought to be four mummies, were “matched.” Almost all of the bone and soft tissue fragments could be relocated correctly, leading to a much more complete appearance of the bodies. This “matching” led to a new total of five individuals. Body C1 consists of an isolated head as well as thorax and lower parts of the body. Body C2 is almost complete yet separated in four major parts (head, upper body, and two legs); its legs are still partially wrapped. Body C3 is headless but thorax, and the majority of the left arm and both legs are in fragmented form preserved. Body C4 is almost complete, except for the feet and hands mostly fragmented. Body D consists of the pelvic girdle and most likely the head of the same individual. Thus, all bodies have suffered major postmortem damage and do not show by naked eye any inscriptions or amulets. The position of the arms of the mummies varies (Table 1).

### 3.2. Mummy C1 (Figure 3)

This fragmented mummy is fairly complete. It clearly shows a female with small, albeit deflated, breasts and a female pelvis (subpubic arcus). The individual age seems to be juvenile, maybe up to adult (ca. 18–25 yrs). Both arms lie straight along her torso; yet the hands have been broken off. The body is now in several components: head (separated from the torso between the seventh cervical and first thoracic vertebra), torso, and legs (the right foot is missing the toes), all of which can be realigned, giving a length of ca. 160 cm (159 cm according to Bach [17], with the maximum length of humerus, as defined by Martin Mass Nr. 1 [18], to be bilaterally 290 mm). The head shows some frontal hacking marks most likely of postmortem nature. The head length is 170 mm and head width is 138 mm. The face is broken, but part of the maxilla and the entire mandible survives, complete with teeth, and the tongue is well preserved too. Twenty-eight teeth are erupted and do not show signs of excessive wear or any dental disease assumed. Some postmortem damage such as a lesion of the right lower first molar and in the frontal part of the left maxilla can be found. The right side of the chin is nicked by a blade. A few wrinkles are visible on the remaining left side of the face.

Unfortunately, the damage makes it impossible to determine if excerebration took place nasally, although from the remaining anatomical elements excerebration looks rather unlikely. Rests of meninges are visible intracranially. The head is covered by short, fine, black, silky hair, with one lock of lighter coloured hair, possibly the result of an excess of natron. The ears are plugged with linen tamped in with resinous material. The body was eviscerated and the abdomen and the pelvic area are stuffed with dense embalming packing materials. The body was wrapped in several layers of linen; the legs and arms are packed separately; much of the bandaging has been removed later. The belly was hacked open and the interior packing was sliced by a sharp blade as is evidenced by cut-marks. The interior cavity is full with packages of bandages that are blackened, presumably by oils and resins. On the back, in the shoulder areas there are also signs of slashing. Based on the healthy teeth, an early adult age can be assumed.

The X-rays show that the head has been separated at the level of the seventh cervical and first thoracic vertebra. A big skull lesion with fragments in the posterior skull cavity can be seen; also parts of the maxilla are separated. Both epiphyses of the iliac bone crest are not fully closed.

The right hand is disarticulated, and of the left hand only three fingers are left; one of it with a fracture in the proximal phalangeal and metacarpal bone. The left ulna and radius are fractured. The left fibula head is most likely fractured too and the former proximal epiphyseal plate is still slightly visible. A fracture at the right lower limb is visible; also the left foot is disarticulated at the upper ankle joint; the distal phalanges of the first toe as well as the middle and distal phalanges of the fifth toe are missing. A subluxation can be found at the calcaneocuboid joint. All these traumata seem to be of postmortem nature. On the conventional X-ray, the thorax shows dense packing in the right part, but no clear signs of remnants of a heart or other mediastinal or pulmonary tissues can be found. The symphysis pubis is hardly visible due to the superimposition of the stuffing material located in the small pelvis; yet specifically both medial menisci are clearly visible.

### 3.3. Mummy C2 (Figure 4)

The head is separated from the torso. The legs, with feet attached, are also separated from the body, with the pelvis attached to the legs. The clavicles are pushed up and the humeri are squashed into the body. The upper arms lay along the body, and the lower arms are moved in so that the hands rested over the pubes. The belly area is broken postmortem, and the left hand is missing, as are portions of the left distal foot.

The remnants of the brain seem to be left in the cranium. Parts of the head, especially the face, are still well wrapped in linen bandages, with the back of the head and hair remaining exposed.

Postmortem cut-marks are visible in the facial bandages on the right side and these are at least four to five centimeters deep. The right eye is sunk in and the bandages are missing, whereas the left eye is fully covered by bandages. The back and top of the head are partially covered by hair that is braided and does not appear to be a wig but the deceased's own hair, although it is possible that some of the braids are woven into the natural hair. The crown of the head is solidly matted, as if oil had been placed there and dribbled into the hair. The ears, however, might have been plugged with linen as the ear openings are distended. The thoracic cavity is completely stuffed with a granular material. In the left thorax region, there is a small soft tissue defect visible. There is a complete separation of the body at the level of the fourth lumbar vertebra.

The robbers have also hacked the body in the belly area and removed the abdominal wall, rendering visible the fact that the body cavity is densely stuffed with rolls of linen impregnated with resinous material. Some sand and gravel are also visible in the body cavity.

The metacarpal bones are present on the right side, whereas for the rest of the left and right side the fingers and wrists are completely missing. Each arm is individually wrapped, spirally, as are the legs. One can count at least a dozen of layers of bandages on the right arm, although originally there were probably more. The legs were similarly wrapped, with the left thigh being wrapped in dozens of layers of linen. The right leg seems to be ca. 1-2 cm longer than the left one; however, this is most likely related to postmortem positioning. Blade marks made by a very sharp implement can be seen in the compacted wrappings of both thighs. The toes are wrapped individually although they are in fewer layers of bandages—two or three—and then wrapped together with the rest of the foot.

On the conventional X-rays, well pneumatized frontal sinuses, a soft tissue defect at the right neck area, and a radio-dense structure of unknown nature at the left ear can be seen. In the right upper thoracic aperture, a radio-dense structure consisting of two parts can be found. A fracture of the right first rib is also visible; also the left 11th rib is broken, most likely postmortem. The iliosacral joint is also most likely fractured postmortem and the left iliac crest is not yet fused. A radio-dense structure of unknown nature can be found between the trapezoid bone and the first metacarpal bone of the right hand.

The stature* in situ* measures ca. 156 cm. However, based on the measurement of humerus, radius, and tibia [19] an average of ca. 165 cm can be assumed—rather a dramatic difference. Based on the pelvis morphology, this is rather a female individual, whereas the skull shows a slight masculine tendency. Its age is most likely young adult, most long bone epiphyses seem fused, and the teeth show a rather low degree of abrasion; yet both iliac crest epiphyses are slightly visible (ca. 20–25 yrs). On the whole, it is more likely to be a male individual.

### 3.4. Mummy C3 (Figure 5)

This mummy is a fairly complete body, although the head and right foot are missing and some extremities damaged. Multiple, uncountable layers of linen, at least four centimeters deep/thick, cover the body. The arms were crossed over the chest, with the left fingers II–V being flexed, with a straight thumb as if it were holding an object. All extremities are multiply broken and the abdomen is exposed. Some soft tissue is missing in the left leg. It is most likely a juvenile to adult individual; most of the epiphyses seem to be closed (ca. 18–25 yrs). The sex is difficult to determine from the pelvis: while the arc composé is rather female, the incisura ischiadica major is indeterminate, and the pelvis in general seems to be rather male. Also, there are no breasts visible. The right humerus length is ca. 345 mm, the tibial length bilaterally each ca. 390 mm; thus, based on Breitinger [19], this would represent ca. 175 cm in total height, more in keeping for males.

The X-rays reveal a.o. a proximal left humerus fracture of most likely postmortem origin. The presence of mediastinal tissue, particularly the heart, cannot be determined due to the filling of the majority of the thorax and abdomen with rather dense stuffing material particularly in the lower abdomen and pelvis. Multiple fractures and anatomical dislocations can be seen: in the left distal lower arm and in the left subtrochanteric region as well as in the left tibia condyles. In the right axillar region, a discontinuity with a soft tissue defect in the humeral head region can be found; as differential diagnosis, a nondislocated humeral fracture of most likely postmortem origin as well as an artefact due to the superimposition of a soft tissue lesion is most likely. Also, the right first metatarsal shows a possible postmortem fracture. Finally, a mildly scoliotic upper thoracic spine toward the left side mostly due to positioning can be seen.

### 3.5. Mummy C4 (Figure 6)

Fairly complete mummy, yet several fragments, parts of feet, and particularly the right arm are missing. It seems to be an adult individual of unknown sex, with a rather female pelvis shape; however, based on secondary sex characteristics (clear absence of female breasts), this is the broken up body of a man, although there is no male genital visible at all. The stature* in situ* is ca. 154 cm. Based on the long bone lengths (length of femur bilaterally ca. 380 mm, medial tibia length right ca. 345 mm, and left ca. 340 mm), the total stature according to Breitinger [19] for a male would be ca. 160 cm.

The partially fully exposed head has thin hanks of hair attached to it, some of which, on the right side, are fairly long. The right ear is crinkled and does not look as if it was pierced. The nose is flattened and there is no indication that it was ever filled with linen plugs to keep its shape. The maxilla shows that all teeth but the third molars had erupted. The teeth show signs of wear and some, particularly the frontal ones, are altered postmortem but no obvious disease is apparent. The mandible is missing. The vast majority of the prevertebral throat area is completely missing.

Only the proximal half of the right humerus survives, and the right radius and ulna are missing. The left arm is broken but present. The upper arms lay along the side of the body; the hands seem to have been placed over the pubes but this is difficult to ascertain due to the mummy's broken state. Both shoulders are positioned cranially (shoulder elevation). The evisceration was in the left lumbar region (ca. 8 cm long), with the cut looking fairly vertical (ca. 15° proximally oriented towards lateral), which might indicate that this mummy was made prior to the end of the reign of Thutmose III (1479–1425) [20]. However, the cut is not clear enough to be completely confident of this dating. The body cavity was filled. The robbers who are responsible for the destruction of the body also cut out a piece of flesh just above the left buttock apparently with a sharp implement.

The X-rays show an unidentifiable small bone fragment at the left femur condyles and a fractured fibula collum as well as a soft tissue defect. The left clavicle is fractured. Also, a massive soft tissue defect right medial in the area of the adductor muscles as well as a unique contour of the femurs bilaterally up to the condyle region can be found. Also, left-sided defects of the pubic bone and symphysis can be seen. All traumata are most likely of postmortem nature. Finally, the massive thoracic (with the exception of the left apex area) and abdominal stuffing can be seen bilaterally.

### 3.6. Mummy D1 (Figure 7)

This body is highly fragmented consisting of a fractured head, the majority of the cervical and thoracic spine, parts of the shoulder blades, the sternum, the proximal part of the right humerus, the pelvis girdle, and major parts of the lower limbs only. This highly tentative grouping is also based on the fact that the legs and pelvic bones definitively match, and this is the only spare head left in the tomb (which from visual inspection may match too). The bandages are virtually all stripped off from the head and the legs.

The frontal part of the head is badly damaged, but there are no clear indications of excerebration via the ethmoid bone as it seems to be intact. The brain as well as the falx cerebri is visible intracranially; also most likely remnants of the brain have been identified in the conventional radiograph (see below). The hair is short and reddish; the color might be the result of ageing or bleaching due to embalming agents. The ears were plugged with linen soaked in resin. The eyes are closed and some of the eyelashes survive. Some natron, the substance which was identified by visual testing, is visible on the occipital area and on the left and right sides of the head and neck, below the jaw line. The following teeth are preserved (Fédération Dentaire Internationale (FDI)/World Dental Federation Notation, ISO-3950 Notation): 13 with postmortem damage, 14 ditto, 15, 16, 17, 22, 23, 24, 25, 26, 27; 35, 36, 37, 46, and 47. They show some degree of abrasion.

The right leg still has some flesh and skin covering the bone, with a few straggling bandages remaining. It is preserved with the exception of the middle part of the femur. The bandages closest to the skin on both legs are dark with oils and resins. The ones further from the skin are brown-beige. The left leg is better preserved. It is possible that part of the genitals (portion of the penis) is preserved, but the soft tissues are difficult to identify, partially due to postmortem damage. The legs show that the individual was probably rather plump during his lifetime as the flesh was folded over at various places.

An inconclusive alteration of the left iliac fossa (area of origin of iliac muscle) can be seen; possible differential diagnoses include those of taphonomic origin (crusts due to water/sand) or to be a periosteal reaction such as a calcified hematoma, though the latter is visually unlikely.

The remnants appear rather female, but it is quite uncertain. It is an adult individual of unknown age, based on the degree of dental alterations most likely within the adult age group (ca. 20–30 yrs). The left medial tibial length is ca. 340 mm; thus, an estimated stature of ca. 155 cm [17] can be assumed for a male individual.

The X-rays show a.o. the cervical spine to be preserved up to the 6th cervical vertebra. A massive soft tissue lesion can be found frontally, with particularly a fractured upper jaw. A fracture and impression of the frontoparietal bones with some bony parts within the skull can also be found. All traumata seem to be of postmortem nature. In the posterior part of the skull, one finds an inhomogeneous substance with no obvious fluid levels; these are most likely remnants of the brain.

## 4. Discussion

Despite being unnamed and only loosely dated, the human remains from KV 31 are useful and significant subjects of study as they provide insight into the mummification practices of mid-18th dynasty elite individuals and into the turbulent later history of the necropolis. The various intense looting phases hamper, however, the analysis of the mummies. The unwrapping of the bodies as well as much of the damage is most probably due to the activity of retrieving valuables and wood at the beginning of the Third Intermediate Period, whereas the scattering of the body parts and the theft of specific mummified body parts (head, hands) can presumably be attributed to robbers of the 19th century AD who sold these pieces to the early tourists. As stated above, these bodies must belong either to members of the royal family or to elite individuals who were in personal contact with the pharaoh. Based on the number of large storage jars found in the tomb, it can be assumed that all five individuals were originally buried here.

All the bodies were carefully mummified, being eviscerated, well desiccated, anointed with oils, and then wrapped with generous amounts of linen. Due to the destructive activities of robbers, one cannot determine the exacte position of the evisceration incision on most of the mummies.

The arm positions for all those whose arms seem to follow the traditional division that was common throughout the New Kingdom and into the Third Intermediate Period, if not beyond: along the sides for women, and over the pubes for men. There is, however, one exception, mummy C3, whose arms are crossed over his chest, with the surviving hand posed as if it had been gripping something. This pose, from the mid-Eighteenth dynasty to the end of the New Kingdom was regularly applied for kings; it became more frequent in later periods. It is, however, still difficult to relate arm positions of mummies to a specific social status or historical period with any degree of certainty. There is, for example, hardly any information available concerning the arm position of royal sons in the 18th dynasty. Also sexing and ageing is difficult to assess due to the partial destruction (and in some cases even crucial missing body parts) and the limited quality of conventional X-rays as well as the superimposition of embalming-related artifacts. Thus, the data need to be taken with enormous caution for these individual criteria. However, in general the bodies seem to be all of adult age. A higher-quality diagnostic imaging approach such as, for example, by CT scanning shall help to better determine individual sex and age and allow an improved evaluation of individual health and disease; the goal of the current excavation project is to get approval for such an advanced logistically more challenging imaging attempt in one of the future field seasons.

The lack of clear medical diagnosis is caused by various factors. Some of the human remains are still wrapped and thus any macroscopic investigation of tissues is impossible. Also, the enormous postmortem damage caused by tomb robbers and the numerous subsequent fractures make a clear distinction of pre- and postmortem origin of the numerous skeletal lesions de facto impossible. Finally, more sophisticated examinations methods were not available* in situ*.

Future studies shall hopefully include ancient DNA analyses as well as C^14^ dating of some of these mummies to possibly match them with existing New Kingdom royal mummy data (e.g., [21]). Especially, a more precise reconstruction of embalming techniques may help to set these individuals into a more exact historical context. Finally, the hereby-described unique human remains show again that the famous Valley of the Kings is still “not exhausted” and may also in the future reveal more insight into ancient life conditions and funerary customs.

## Figures and Tables

**Figure 3:**
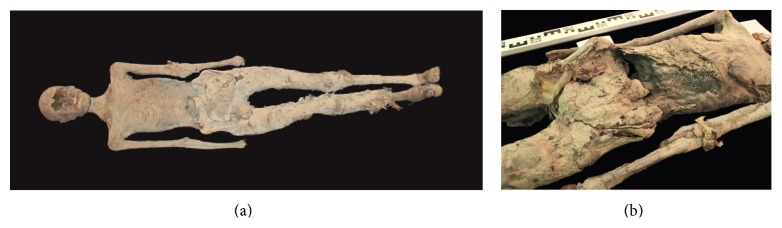
(a), (b): overview of mummy C1 (a) and close-up view of postmortem exposed abdominal cavity showing packages of bandages (b).

**Figure 4:**
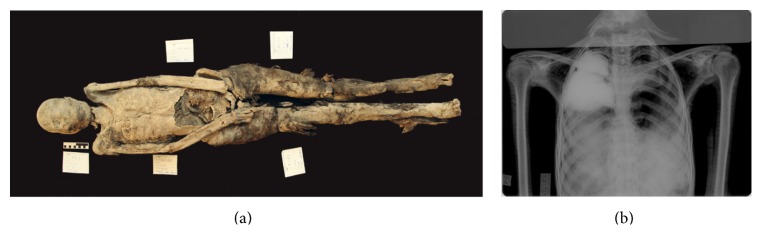
(a) overview of mummy C2, (b) Conventional x-ray (ap direction) of thorax showing among others in the right upper thoracic cavity two dense structures most likely to be organ or other packages.

**Figure 5:**
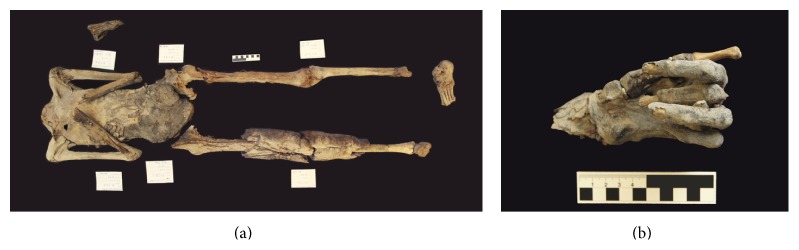
(a), (b): overview of mummy C3 (a), close-up of left hand with flexed fingers II–V (b).

**Figure 6:**
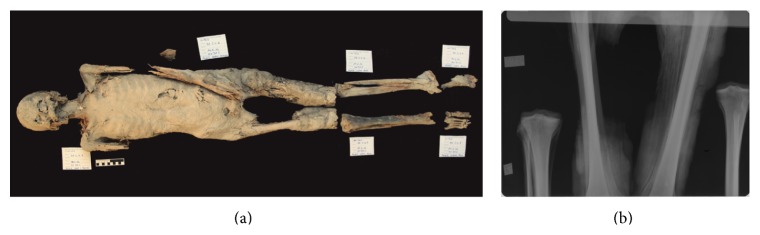
(a), (b): overview of mummy C4 (a) and conventional X-ray (ap direction) of midfemoral region (with parts of isolated lower limbs visible too) showing a.o. the massive soft tissue defects in the region of the adductor muscles.

**Figure 7:**
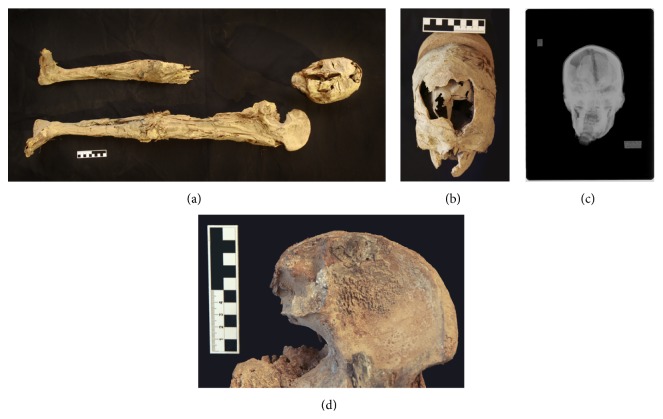
(a), (b), (c), and (d): overview of mummified remains D1 (a), frontal view of head D1 (b), conventional X-ray of head D1 showing among others most likely remnants of the shrunken brain (c), and close-up of alteration in left iliac fossa region of unclear etiology (d).

**Table 1:** Basic description of mummies investigated (n.d.: not determinable).

Mummy	Sex	Age	Height (cm)	Eviscerated	Excerebrated	Position of arms	Disease (besides unhealed fractures)
31.C.1	F	Juvenile-young adult	155–165	Yes	Probably not	Side	None
31.C.2	M?	Young adult?	Ca. 165	Yes	Probably not	Pubes	None
31.C.3	M?	Juvenile-young adult	Ca. 175	Yes	No skull preserved	Crossed	None
31.C.4	M?	Adult	n.d.	Yes	n.d.	Pubes	None
31.D.1	F?	Adult	Ca. 155	n.d.	Certainly not	n.d.	Unclear finding in iliac fossa
